# Uterus didelphys complicated with endometrial carcinoma: A case report of uterus didelphys with endometrial carcinoma

**DOI:** 10.1097/MD.0000000000029183

**Published:** 2022-05-13

**Authors:** Liang Chen, Fang Zhang, Yue-bing Ma, Jin-long Chen

**Affiliations:** a Postdoctoral Research Station, Tianjin Medical University, Tianjin, P.R. China; b Department of Gynecological Oncology, Shandong Cancer Hospital and Institute, Shandong First Medical University and Shandong Academy of Medical Sciences, Jinan, P.R. China; c Department of Radiology, Provincial Hospital Affiliated to Shandong First Medical University, Jinan, P.R. China.

**Keywords:** endometrial cancer, laparoscopy, uterine didelphys

## Abstract

**Rationale:**

The incidence of uterine malformations is low (4%–7%). Currently, the National Comprehensive Cancer Network clinical practice guidelines in oncology recommend minimally invasive surgery for early endometrial cancer. Minimally invasive surgery for the treatment of uterine didelphys with endometrial cancer is rare due to the large size of the uterus. To date, only 2 such patients have been reported to have undergone laparoscopy. Whether such patients can be treated with minimally invasive surgery needs to be further explored.

**Patient concerns:**

A 40-year-old woman with uterine didelphys was hospitalized for menorrhagia in the past 2 months.

**Diagnosis:**

Endometrial adenocarcinoma was found in both the uterus and cervix using fractional dilation and curettage.

**Interventions:**

The patient underwent laparoscopic surgery. Postoperative adjuvant radiotherapy and chemotherapy were administered.

**Outcomes:**

There was no sign of recurrence during routine follow-up.

**Lessons:**

The use of a uterine manipulator to lift either side of the uterus could help to expose the narrow ipsilateral para-uterine field. It is difficult to remove the uterus entirely through the vagina, making it necessary to select appropriate cases wherein screening is performed to check if the vagina is loose, and the uterus is of appropriate size. Minimally invasive surgery may be feasible for suitable patients.

## 1 Introduction

The overall incidence of uterine anomalies is low and the clinical diagnosis of a specific malformation is often difficult and confusing.^[1]^ Uterine didelphys may often be misdiagnosed as bicornate/mediastinal uterus. Uterine didelphys occur when the bilateral Müller tubes completely fail to become proximal and fuse with each other and instead continue to develop individually, thus forming 2 uterine cavities, 2 cervixes, and often 2 vaginas separated by the longitudinal diaphragm and with normal menstrual flow. A bicornuate uterus occurs when the fusion of 2 Müllerian structures fails, resulting in 2 uterine horns and only 1 cervix. The mediastinal uterus is the result of insufficient reabsorption of the septum after fusion of Muller's structure.

In developed countries, the most common malignant tumor of the female reproductive system is endometrial carcinoma (EC). In China, its incidence is second only to cervical cancer but shows a significant upward trend. Randomized controlled trials and meta-analyses have shown that compared to laparotomy, laparoscopy, a minimally invasive treatment, offers the benefits of a lower incision infection rate, lower blood transfusion rate, lower venous thrombosis incidence, shorter hospital stay, higher quality of life, and no difference in tumor-related prognosis. Therefore, laparoscopy is the primary recommendation of the National Comprehensive Cancer Network clinical practice guidelines in oncology for early endometrial cancer.^[2–8]^ Uterine didelphys complicated by EC are rare. Furthermore, enlargement of uterine didelphys makes the use of minimally invasive surgery even more challenging. To date, only 2 cases involving the use of minimally invasive surgery for uterine didelphys have been reported. Here, we report a case in which laparoscopy was successfully performed for uterine didelphys complicated by EC.

## 2 Case report

**Figure 1:**
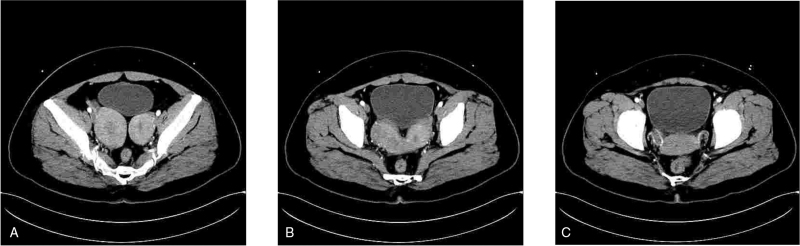
CT findings of the uterus. (A) CT shows bilateral enlargement of the uterine body. (B) Bilateral uterine fusion and adhesion to the bladder wall. (C) Bilateral cervix and bilateral uterine arteries. CT = computed tomography.

A 40-year-old woman was hospitalized for menorrhagia over the previous 2 months. She did not have any other diseases and had no particular family history. The patient was married and had a childbearing history of G0P0L0A0. Computed tomography showed uterine didelphys, thickened endometrium in the uterine and cervical canals, mild-to-moderate enhancement in the right endometrium, and small lymph nodes in the abdominal cavity and retroperitoneum (Fig. 1). Gynecological examination revealed a normal vulva and urethral orifice, an unobstructed vagina, and smooth mucosa. A double cervix was observed above the vagina and both cervices had a diameter of approximately 2 cm. The pathology of fractional dilation and curettage was as follows: left uterine cavity, moderately to highly differentiated endometrioid carcinoma; right uterine cavity, moderately differentiated endometrioid carcinoma; left cervical canal, well-differentiated endometrioid carcinoma; and right cervical canal, moderately to highly differentiated endometrioid carcinoma. Two diagnoses were established, EC and uterine didelphys.

**Figure 2:**
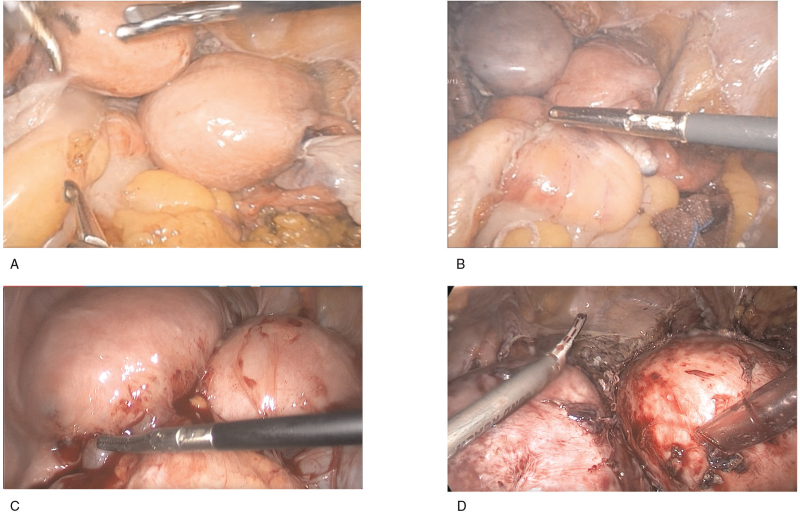
Laparoscopic findings and intervention. (A) Laparoscopic view of the bilateral uterus, each with an adnexal fallopian tube and ovary. (B) Ischemic discoloration of the left uterus after cutting off both sides of the pelvic funnel ligament and the left uterine artery. (C) Laparoscopic view of the bilateral uterus. (D) Ischemic discoloration of the left uterus after cutting off the right pelvic funnel ligament and the left uterine artery.

**Figure 3:**
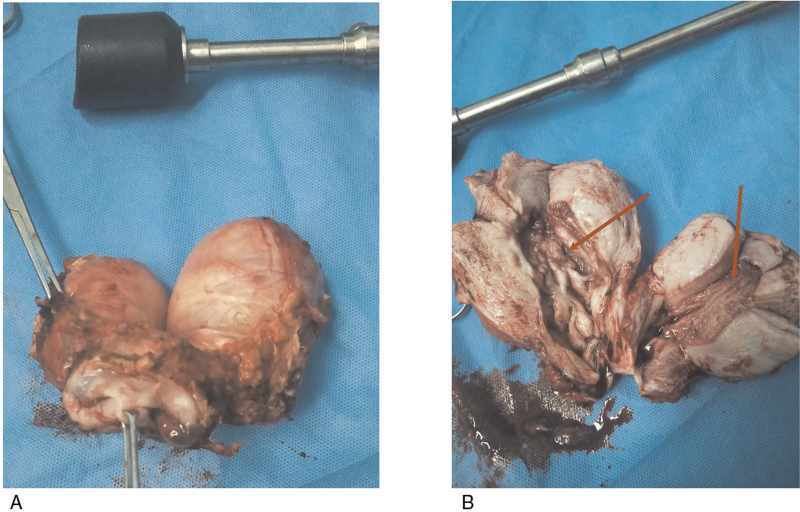
Resected specimens and observations. (A) Gross specimen display. (B) Dissected specimen, cancer foci can be seen in the uterine cavity of the double uterus (as shown by the arrows).

On March 23, 2018, surgery was performed under general anesthesia. Fractional dilation and curettage before surgery indicated that both the uterus and cervical canal showed cancer involvement; therefore, modified radical hysterectomy was performed. The patient underwent laparoscopic modified radical hysterectomy, double adnexal resection, pelvic lymphadenectomy, and abdominal para-aortic lymphadenectomy (Fig. 2). Postoperative gross pathology findings were as follows (Fig. 3): the volume of the right uterus was 10.5 × 8 × 4.5 cm. The area in the uterine cavity showing grayish-red erosion was approximately 6 × 4 cm. The volume of the left uterus was 9 × 7 × 4 cm, and the rough endometrial surface area was grayish-white or grayish-red, measuring approximately 4 × 2 cm. Routine pathological diagnosis was as follows: the right uterus showed a differentiated endometrioid adenocarcinoma, which invaded the superficial myometrium and involved the internal orifice of the cervical canal. The left uterus showed differentiated endometrioid adenocarcinoma invading the superficial myometrium but not the internal orifice of the cervical canal. The cervix showed bilateral, chronic mucosal inflammation. No lymph node metastasis was observed. The immunohistochemical status was 70% estrogen receptor (+) and 70% progesterone receptor (+).

A postoperative pathological diagnosis of stage I and G2 endometrial adenocarcinomas was made. Postoperatively, paclitaxel + carboplatin combination therapy was administered for 4 cycles simultaneously with afterloading radiotherapy. The patient was routinely followed up by telephone in December 2021. She underwent computed tomography examination in 2020, wherein she showed no signs of tumor recurrence or obvious complications.

The findings during the operation were as follows: The space between the uterine isthmus/cervix and the posterior wall of the bladder was dense and unclear. The uterus and bladder were separated to expose the cystocervical space mainly via sharp separation. Since both uterine cavities are large and occupy the majority of the pelvic operation field, it is difficult to lift the bladder and expose the para-uterine space. The left uterus was lifted to expose and resect the left parauterine tissue and the right uterus was lifted to expose and remove the right uterine tissue. This effectively solved the problem of limited visual field exposure. After resection of the bilateral pelvic funnel ligaments, only the left uterus became ischemic and discolored when severing the left uterine artery. This suggests that unilateral uterine arteries supply only the uterus along their corresponding sides. After complete resection of both uteri, removal of uterine didelphys through the vagina was challenging. Thus, we deflected the uterus and first pulled out the left uterine horn of the left uterus, removed the small uterus on the left, and successfully removed the larger uterus on the right.

## 3 Discussion

The incidence of uterine malformations is low (4%–7%),^[9,10]^ and there is no evidence that uterine malformations lead to an increase in the incidence of EC. As fractional dilation and curettage revealed endometrioid adenocarcinoma in both uteri, complete resection of both uteri was performed. We reviewed published articles on uterine malformations complicated by endometrial cancer in PubMed from January 1, 1990 to June 30, 2021, and screened the articles on double uterine malformations. We found only 18 cases of uterine didelphys complicated by malignant tumors, including 15 cases of EC, 2 cases of carcinosarcoma, and 1 case of sarcoma.^[9,11–27]^ The incidence of bilateral uterine involvement was even lower, with only 3 cases reported in the literature, including 1 case of sarcoma. In our patient, diagnostic curettage revealed involvement of both the uterine cavity and cervix; therefore, modified radical hysterectomy was chosen. For uterine didelphys, diagnostic curettage should avoid missing 1 side of the diseased uterus, which may result in missed diagnosis. Patients with uterine didelphys often present with double vaginas, and special attention should be paid to identifying exceptions. In patients with bilateral cervical involvement, cervical tube curettage should be performed separately to avoid missing lesions.

The use of laparoscopy, because of its minimal invasiveness, is advantageous in EC and offers a prognosis comparable to that of laparotomy. It is also associated with lesser trauma, faster recovery, and fewer intestinal adhesions, making it superior to open surgery. Our patient was satisfied with the minimally invasive surgery and had a short postoperative recovery period. After a thorough literature review, we found that only 1 case each of robotic and laparoscopic surgeries has been reported.^[13,26]^ This may be attributed to the uterine didelphys being generally large and thus difficult to remove through the vagina. Despite the challenge of the enlarged uterus, we pulled it out completely through the vagina. This proves that minimally invasive surgery can be performed after screening to determine whether the vaginal fornix is loose. In our case, there were some complications with laparoscopy: both uteri were large, the field of vision was narrow and difficult to expose, and lifting the uterus was inconvenient. However, a uterine manipulator was used to lift 1 side of the uterus and move it toward the opposite side to expose and remove ipsilateral para-uterine tissue. This could effectively solve the problem of exposure difficulties in the bilateral para-uterine fields. Recently, we performed laparoscopic hysterectomy in another patient with uterine didelphys and only 1 kidney. The patient was married and had a childbearing history of G1P1L1A0. This patient had adenomyosis in both uteri that was complicated by endometriotic cysts in both ovaries. The patient experienced severe dysmenorrhea and required surgery to relieve her symptoms. During the surgery, the visual field was as difficult to expose as in the previous patient. We used the above-mentioned method to solve this problem, which was using uterine manipulator to lift 1 side of the uterus to expose the ipsilateral para-uterine field.

During the surgery, we found that 1 side of the uterine artery was supplied to only 1 side of the uterus in this patient with uterine didelphys. In young patients wishing to retain reproductive function, if only 1 side of the uterus is affected by endometrial cancer, it may be possible to cutoff the artery supplying the affected side and resect the affected side of the uterus. Jiao et al reported a 28-year-old infertile woman with uterine didelphys who tried to preserve the uterus even after the failure of megestrol treatment. The patient underwent transabdominal surgery to remove the left uterus and fallopian tube, thereby preserving the contralateral uterus and appendages. This relationship was also verified in another patient with uterine didelphys. This preliminarily confirms that the embryonic stage of the uterine didelphys is formed separately, and that it is feasible to retain 1 side of the uterus and uterine artery.^[25]^

Minimally invasive surgery has the benefits of reduced trauma and a faster postoperative recovery. To check if patients with uterine malformations are eligible for minimally invasive surgery, we suggest that the following conditions should be met: the vaginal fornix should be loose and the uterus should be of appropriate size. Understanding the different anatomical characteristics and careful cooperation of the entire team are also warranted.

## Author contributions

**Data curation:** Jin-long Chen, Yue-Bing Ma.

**Formal analysis:** Fang Zhang, Liang Chen.

**Funding acquisition:** Liang Chen.

**Investigation:** Fang Zhang, Jin-long Chen, Yue-Bing Ma.

**Writing – original draft:** Fang Zhang, Jin-long Chen, Liang Chen.

**Writing – review & editing:** Liang Chen.
